# Fifty-year habitat subdivision enhances soil microbial biomass and diversity across subtropical land-bridge islands

**DOI:** 10.3389/fmicb.2022.1063340

**Published:** 2022-12-09

**Authors:** Ying Wu, Bing Wang, Liji Wu, Shengen Liu, Lingyan Yue, Jianping Wu, Dima Chen

**Affiliations:** ^1^Yunnan Key Laboratory of Plant Reproductive Adaptation and Evolutionary Ecology, Yunnan University, Kunming, China; ^2^Key Laboratory of Soil Ecology and Health in Universities of Yunnan Province, School of Ecology and Environmental Science, Yunnan University, Kunming, China; ^3^Engineering Research Center of Eco-Environment in Three Gorges Reservoir Region of Ministry of Education, China Three Gorges University, Yichang, China

**Keywords:** community assembly, habitat subdivision, mainland-island system, island biogeography, soil microbial diversity, habitat heterogeneity

## Abstract

Although habitat loss and subdivision are considered main causes of sharp declines in biodiversity, there is still great uncertainty concerning the response of soil microbial biomass, diversity, and assemblage to habitat subdivision at the regional scale. Here, we selected 61 subtropical land-bridge islands (with small, medium, and large land areas) with a 50-year history of habitat subdivision and 9 adjacent mainland sites to investigate how habitat subdivision-induced unequal-sized patches and isolation affects biomass, diversity, and assemblages of soil bacteria and fungi. We found that the soil bacterial and fungal biomass on all unequal-sized islands were higher than that on mainland, while soil bacterial and fungal richness on the medium-sized islands were higher than that on mainland and other-sized islands. The habitat subdivision-induced increases in microbial biomass or richness were mainly associated with the changes in subdivision-specified habitat heterogeneities, especial for soil pH and soil moisture. Habitat subdivision reduced soil bacterial dissimilarity on medium-sized islands but did not affect soil fungal dissimilarity on islands of any size. The habitat fragment-induced changes in soil microbial dissimilarity were mainly associated with microbial richness. In summary, our results based on the responses of soil microbial communities from subtropical land-bridge islands are not consistent with the island biogeographical hypotheses but are to some extent consistent with the tradeoff between competition and dispersal. These findings indicate that the response of soil microbial communities to habitat subdivision differed by degree of subdivision and strongly related to habitat heterogeneity, and the distribution of microbial diversity among islands is also affected by tradeoff between competition and dispersal.

## Introduction

How habitat loss and fragmentation affects biodiversity and ecosystem functions is an important topic in ecological research (Lindenmayer and Fischer, 2007; Haddad et al., 2015; Si et al., 2016). Empirical studies in macro ecosystem commonly emphasize that habitat loss has triggered negative effects on biodiversity, while the effects of habitat fragmentation *per se* (hereafter termed habitat subdivision) are still large uncertainties (Haddad et al., 2015; May et al., 2019). Habitat subdivision is a landscape-level reconfiguring that subdividing a single large of habitat into several smaller areas involves multidimensional effects on biodiversity (Ewers and Didham, 2006; Lindenmayer and Fischer, 2007). The most intuitive effects are derived from increase of habitat patches, decrease of habitat area, changes of isolation, and even novel ecological boundaries (Ewers and Didham, 2006; Haddad et al., 2017; Bueno and Peres, 2019). It is still controversial to clarify the impacts of habitat subdivision due to its divergent changes in configuration (Villard and Metzger, 2014). The habitat amount hypothesis holds that the effect of habitat subdivision is consistent with the effect of habitat amount changes (Haddad et al., 2017). However, increased researches have demonstrated that the impacts of habitat area reduction and isolation changes caused by habitat subdivision on biodiversity cannot be replaced by the impacts of habitat amounts (Haddad et al., 2017; Deane et al., 2022). Therefore, habitat subdivision induced changes of geometric properties may strongly influence biodiversity and ecosystem functions directly and indirectly (Deane et al., 2022). The geometric effects of habitat subdivision on soil microbial biomass and diversity are especially unclear due to the substantial mismatch between habitat subdivision scale and microbial habitat area or diffusion capacity (Baas-Becking, 1934; Finlay, 2002). Furthermore, few studies have focused on the responses of soil microbial biomass and diversity to long-term (e.g., more than 50 years) habitat subdivision (Peay et al., 2007; Vannette et al., 2016), despite the important contribution of soil microorganisms to ecosystem functions (van der Heijden et al., 2007; Delgado-Baquerizo et al., 2016).

Habitat subdivision may alter soil microbial biomass and diversity at a regional scale by changing patch sizes and isolation, and soil abiotic and biotic factors (Peay et al., 2007; Vannette et al., 2016; Fanin et al., 2018; Li et al., 2020b). It is generally considered that the distribution pattern of microorganisms is completely different from that of macroorganisms due to the high species number, high diffusion ability, and small size of microorganisms (Baas-Becking, 1934; Finlay, 2002; Si et al., 2016; Zeng et al., 2019). Nonetheless, studies have increasingly indicated that unequal-sized patches and diffusion limitation caused by habitat subdivision are main factors affecting soil microbial diversity (Peay et al., 2007; Vannette et al., 2016; Li et al., 2020b). A study conducted in a lava-fragmented landscape, for example, showed that root-associated fungal communities were affected by fragmented area (Vannette et al., 2016), which was consistent with the theory concerning the relationship between species and area relationship based on macroorganisms (Macarthur and Wilson, 1963, 2016). Researchers have also suggested that changes in soil microbial biomass and diversity after habitat subdivision could be explained by other theories, such as island biogeographical theory (Macarthur and Wilson, 1963, 2016) and the regional similarity hypothesis (Mouquet and Loreau, 2002), which states that community changes result from the interaction between dispersal and species competition. In addition, the changes in the soil environment and biotic interactions caused by habitat subdivision can also greatly affect soil microbial diversity (van der Heijden et al., 2007; Fierer, 2017; Li et al., 2020b). The area of fragmented habitat, for example, can affect soil bacterial diversity through soil moisture (Li et al., 2020b) and can affect root-associated fungal communities through their host plants (Vannette et al., 2016). Landscape ecologists have therefore suggested that future habitat fragmentation studies should assess the effects of unequal-size patches and isolation (Wilson et al., 2016).

Explaining the effects of habitat subdivision on species and communities is difficult because of the ecosystem differences in connectivity, habitat area, and heterogeneity (Si et al., 2016; Wilson et al., 2016; Zeng et al., 2019). For example, habitat subdivision studies often involve hidden uncertainties related to incomplete diffusion barriers (e.g., corridors) or large-scale matrix changes (e.g., fire and logging); such uncertainties make it difficult to generalize about the effects of habitat subdivision on biodiversity (Wardle et al., 1997; Haddad et al., 2015; Wilson et al., 2016; Kardol et al., 2018). Simplified experimental studies, such as mesocosm or microcosm experiments (Åström and Bengtsson, 2011; Delgado-Baquerizo et al., 2018), cannot provide a complete understanding of the natural response of biodiversity to habitat subdivision. As an alternative to mesocosm and microcosm experiments, islands provide a more natural system for studying basic problems of ecology and evolution (Wardle et al., 1997; Losos and Ricklefs, 2009; Kardol et al., 2018), and land-bridge islands in particular are ideal model systems for testing the consequences of habitat subdivision (Si et al., 2016; Wilson et al., 2016; Zeng et al., 2019). In the case of land-bridge islands, the fragmented habitats are surrounded by water with a high-contrast heterogeneity, which severely limits the dispersal of certain taxa (Wilson et al., 2016). On the other hand, land-bridge island system is also a natural laboratory for verifying theory of island biogeography (Wilson et al., 2016; Li et al., 2020b). However, only a few studies have explored the distribution patterns of soil microorganisms among islands (Li et al., 2020b). Larger islands may lead to higher soil microbial diversity due to higher soil environmental heterogeneity, however, there is still doubt whether landscape-scale habitat heterogeneity can match the scale threshold of soil microorganisms (Bru et al., 2011; Fierer, 2017).

In the current study, we used mainland-island system to explore two questions: (1) examine effects and process of unequal-sized patches and isolation after habitat subdivision on soil microbial biodiversity in fragmented islands *via* comparing soil microbial biodiversity and composition among mainland fragmented into small islands, mainland fragmented in to medium islands, and mainland fragmented in to large islands; (2) verify island biogeographic theory and heterogeneity hypothesis *via* finding major factors that affect soil microbial diversity and composition among islands. The islands, which are located in Jiangxi Province, China, were created in 1972 when Zhelin Reservoir was established (Supplementary Figure S1). In 2020, after the islands had experienced nearly 50 years of habitat subdivision, we assessed the soil bacterial and fungal communities and soil abiotic variables on 61 land-bridge islands and 9 adjacent mainland sites. We categorized the 61 land-bridge islands into three types based on land area (small, medium, and large), and we also characterized the attributes of each island (e.g., distance to mainland, perimeter to area ratio, and island shape index). We hypothesize that: (1) soil microbial biomass and diversity would be lowest when mainland fragmented into small-sized islands because small patches may be too small to sustain a local population (Debinski and Holt, 2000); (2) soil microbial biomass and diversity may be not suitable for island biogeography theory but support other theories such as habitat heterogeneity hypothesis because of the mismatch between fragmented scale and micro-habitat scale (Sonnier et al., 2014) or dispersal-competition tradeoff (Mouquet and Loreau, 2002).

## Materials and methods

### Study sites

The Zhelin Reservoir is located at 29°03′-29°18′N and 115°04′-115°40′E in northwest Jiangxi Province, China (Supplementary Figure S1). In 1972, the Zhelin Reservoir was created by the damming of the middle reaches of the Xiushui River; with the rise of water level, thousands of islands were created in the reservoir (Lu et al., 2013). The Zhelin Reservoir is dominated by a subtropical monsoon climate, and subtropical evergreen broadleaf forest is the native vegetation. The mean annual precipitation ranges from 1,461 to 1,582 mm, and the mean annual temperature ranges from 17.3 to 17.5°C. In December of 2021, about 50 years after reservoir establishment, we selected 61 land-bridge islands and 9 mainland sites with minimal human interference. The elevation of islands and mainland sites ranged from 56 to 135 m above sea level (mean water depth is above 55 m), and the dominant tree species are *Cyclobalanopsis glauca* and *Pinus massoniana*. The climatic datasets during 1979–2022 from three stations around our study sites showed that mainland and three types of islands had similar historical climate conditions.

### Measurement of land-bridge island attributes

We digitized the map of the Zhelin Reservoir from Google Earth^™^ and calculated the area and perimeter of all 1,080 islands using ArcGIS 10.2 (ESRI, Inc., Redlands, CA, United States). Based on the position, area, and perimeter of these islands, we randomly selected 61 islands. To explain the effects of unequal-sized patches after habitat subdivision, we classified the 61 islands into three “area types”: small islands (< 1 ha, *n* = 15), medium islands (> 1, < 5 ha, *n* = 31), and large islands (> 5 ha, *n* = 15). The 9 mainland sites were on the north (4 sites) and south (5 sites) riverbanks. The distance of each land-bridge island to the mainland and to the nearest island was calculated in R version 3.6.3 (R Core Team, R, 2017). More specifically, the distance to mainland was calculated from each island to the north or south riverbank (that is distance to nearest coastline) *via* the dist2Line function in the ‘geosphere’ package, and the distance to the nearest island was calculated *via* the gdit function in the ‘Imap’ package. We also calculated the ratio of the perimeter to the area and the island shape index (*S*=*P*/[2 × (*π* × A)]^0.5^) for each island (Yu et al., 2012).

### Soil sampling and analysis and soil microbial community analysis

Soil samples were collected from all land-bridge islands and mainland sites in December 2020. Three evenly spaced, concentric circles from the center to the edge of each island were designated for soil sampling; the radii of the circles (i.e., sampling lines) increased with island size, and the outermost circles were at least 10 m from the island edge. Soil samples were also collected from 9 mainland sites that had minimum human interference and that were located on both sides of the reservoir. At each mainland site, three evenly spaced sampling lines were designated; each line was 50 m long, was perpendicular to the reservoir bank, and extended from the highest to the lowest altitude. For each island and mainland sampling line, we collected five soil samples by taking 7-cm-diameter soil cores in the topsoil (0–10 cm); these were mixed to yield one composite sample per line and three samples per land-bridge island and mainland site. After roots were removed, the fresh soil samples were passed through a 2-mm-mesh sieve. One part of the soil sample was aired-dried and used for analysis of soil pH with a 1:2.5 (soil: water) suspension, soil organic carbon (SOC) using the Walkley-Black modified acid-dichromate FeSO_4_ titration method, total soil nitrogen (TN) using micro-Kjeldahl digestion, and total soil phosphorus (TP) using the H_2_SO_4_-HClO_4_ fusion method (Chen et al., 2016, 2019). The other part of each moist soil sample was used for determining soil moisture and the soil microbial properties.

Soil bacterial and fungal biomasses were estimated by phospholipid fatty acid (PLFA) analysis (Chen et al., 2016, 2019), which were extracted using 8 g freeze-dried soil for each sampling. Biomasses (nmol g ^−1^ of dry soil) were determined according to PLFAs specific to bacteria (i14:0, i15:0, a15:0, i16:0, 16:1ω7c, i17:0, a17:0, cy17:0, 17:1ω8, 18:1ω7c, 18:1ω9, and cy19:0) and fungi (18:2ω6,9). The bacterial to fungal biomass ratio (B:F ratio) was also calculated. For assessment of soil bacterial and fungal diversity and composition, soil DNA was extracted from a 0.5-g soil sample using the FastDNA^®^ Spin Kit for Soil (MP Biomedical, Solon, OH). We targeted the V3-V4 region of bacterial 16S rRNA gene *via* 338F-806R primers and the ITS sequence of the fungal gene *via* ITS1-ITS2 primers using an Illumina MiSeq platform (San Diego, CA, United States). Demultiplexed paired-end fastq files were used to complete downstream processing using QIIME2 with the DADA2 denoising method (Callahan et al., 2016). After sequences were trimmed (to remove adapters) and truncated (to remove sequences with low quality), we filtered amplicon sequencing variants (ASVs) that had <0.01% relative abundance or that only appeared in three samples. The taxonomy of each gene sequence was analyzed by the Naive Bayes Classifier algorithm against the Silva database (Silva 138) for bacteria and the Unite database (Unite 8.2) for fungi (Abarenkov et al., 2020). After rare ASVs and singletons were filtered, 1,512 bacterial ASVs and 1,204 fungal ASVs remained.

### Statistical analysis

First, differences in microbial biomass, alpha diversity, island attributes, and soil abiotic factors among mainland and island area types were examined using Bayesian-ANOVA based on the independent variances jags model *via* ‘rjags’ packages (Kruschke, 2014). The posterior distribution of response variables in each group and the differences between two groups were estimated using Markov Chain Monte Carlo (MCMC) sampling techniques. When the 95% highest density interval (HDI) of the posterior distribution of the difference between two groups fell above or below zero, we concluded that the means of the groups were different (Kruschke, 2014). Second, differences in microbial composition among mainland and island area types were visualized using principal coordinate analyses (PCoAs) from Bray-Curtis distances. Permutational multivariate analysis of variance (PERMANOVAs) with the *adonis* function in the ‘vegan’ packages were used to determine whether bacterial and fungal composition differed among mainland and island area types. CoDA method in ‘Selbal’ package was used to detect microbial signatures (global balance) between mainland and islands (Rivera-Pinto et al., 2018). The correlations between soil properties and relative abundance of dominant bacterial phyla or fungal classes, and microbial signatures were estimated by Pearson correlation analysis. Third, we used structural equation modelling (SEM) to determine the potential direct and indirect effects of long-term habitat subdivision on soil microbial biomass, richness, and dissimilarity *via* response ratio between mainland and islands: (Variable_island_ – Variable_mainland_)/Variable_mainland_. We assumed that habitat fragmentation would first change habitat area and isolation, and indirectly affect habitat heterogeneity, including soil moisture, soil pH, soil nutrient quantity, and soil nutrient quality. Each hierarchical model was simplified by step-wise exclusion of pathways that had non-significant regressions (*p* > 0.05) and was implemented using the maximum likelihood estimation method (Chen et al., 2019). In the SEM, we classified the soil-nutrient explanatory variables into two categories (quantity and quality) based on previous reports (Chen et al., 2016, 2019): (1) soil substrate quantity included SOC, TN, and TP; and (2) soil substrate quality included the soil C:N ratio, C:P ratio, and N:P ratio. The first principal component (PCA1) of two categories explained 66 and 73% of the total variance, respectively. To test island biogeography theory, we used ordinary least squares regression between microbial richness and island area or distance to mainland. Finally, random forest classification analysis and accuracy significance were used to select the most important determinants of microbial biomass, richness, and dissimilarity among island types, and generalized additive models (GAMs) were used *via* the ‘mgcv’ package in R to investigate natural relationships between determinants and microbial biomass, richness, and dissimilarity. The SEM models were developed with AMOS 21 software (IBM SPSS Inc., Chicago, IL, United States). Other statistical analyses were performed using R version 3.6.3 (R Core Team, R, 2017).

## Results

### Responses of land-bridge island attributes and soil abiotic variables

**Figure 1:**
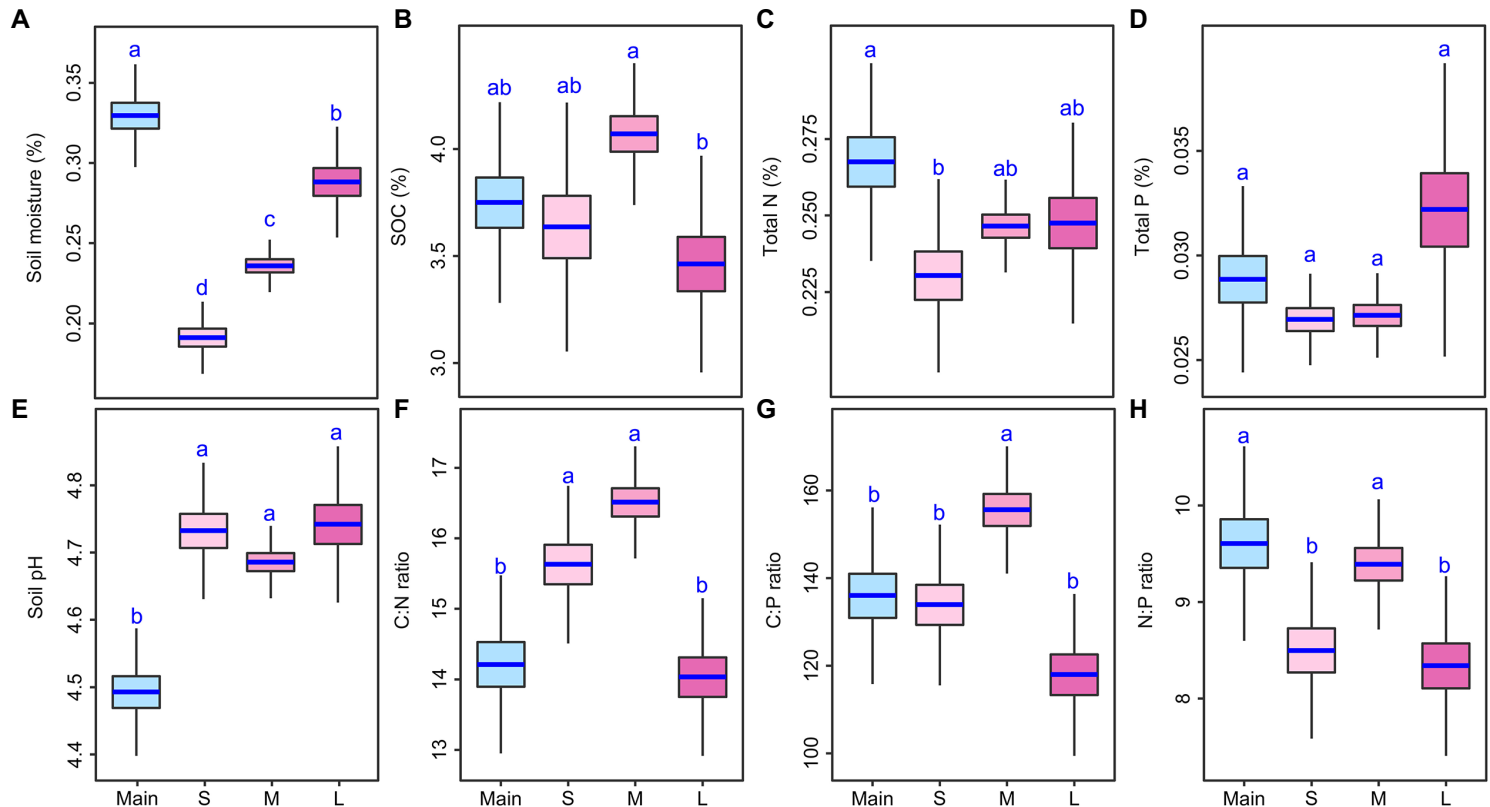
Differences in soil abiotic variables **(A–H)** among the mainland (Main) and three island types (S: small islands; M: medium islands; L: large islands). Boxplots indicate the medians and upper and lower quartiles of the posterior distribution. Different letters indicate a credibly different posterior distribution among the mainland and three island types (Bayesian-ANOVA, 95% highest density intervals of the posterior difference between each two groups falls above or below zero).

Soil abiotic variables substantially differed among mainland sites and the three island-area types (Figure 1; Supplementary Table S1). The soil moisture of islands was lower than that of the mainland sites at least 12% and soil pH of island was higher than that of the mainland sites at least 2 units (Figure 1; Supplementary Table S1). SOC and TP were similar between the mainland sites and each of the three types of islands (Figure 1). The mainland sites had higher TN and soil N:P than small or large islands, but had lower soil C:N and C:P than small or medium islands (Figure 1; Supplementary Table S1). Soil moisture increased, but soil pH, TN, and TP stayed constant as island size increased; SOC, soil C:N, soil C:P, and soil N:P were higher on the medium-sized islands than on small or large islands (Figure 1; Supplementary Table S1). Island area, perimeter, shape index, and nearest distance increased as island size increased, but distance-to-mainland and perimeter: area ratio decreased as island size increased (Supplementary Figure S2, Table S2).

### Responses of soil microbial biomass, richness, dissimilarity, and composition

**Figure 2:**
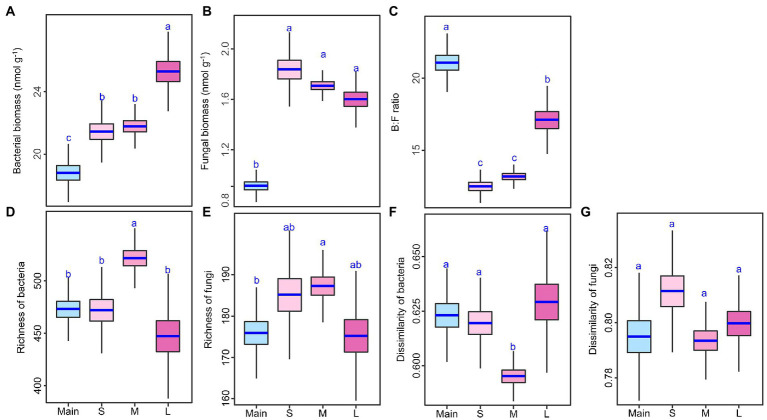
Differences in soil bacterial and fungal biomass, amplicon sequencing variant (ASV) richness, and dissimilarity **(A–G)** among the mainland (Main) and three island types (S: small islands; M: medium islands; L: large islands). Boxplots indicate the medians and upper and lower quartile of the posterior distribution. Different letters indicate a credibly different posterior distribution among the mainland and three island types (Bayesian-ANOVA, 95% highest density intervals of the posterior difference between each two groups falls above or below zero).

Soil bacterial biomass and B:F ratio were higher on the large-sized islands than on the small or medium islands, but soil fungal biomass did not differ among the three sizes of islands (Figures 2A–C; Supplementary Table S3). Compared with the mainland, each type of islands had higher soil bacterial biomass and fungal biomass but had lower B:F ratio. For example, the increase of bacterial biomass on large island reached to 25% and the increase of fungal biomass on small island reached to 50% (Figures 2A–C; Supplementary Table S3). Soil bacterial richness was higher on the medium-sized islands than on the small or large islands, but soil fungal richness did not differ among the three sizes of islands (Figures 2D,E; Supplementary Table S3). Compared with the mainland, however, medium-sized islands but not small or large islands had higher bacterial and fungal richness (Figures 2D,E; Supplementary Table S3). Among the mainland and three types of islands, the medium islands had the lowest bacterial dissimilarity, but fungal dissimilarity did not differ among the mainland and three types of islands (Figures 2F,G; Supplementary Table S3).

**Figure 3:**
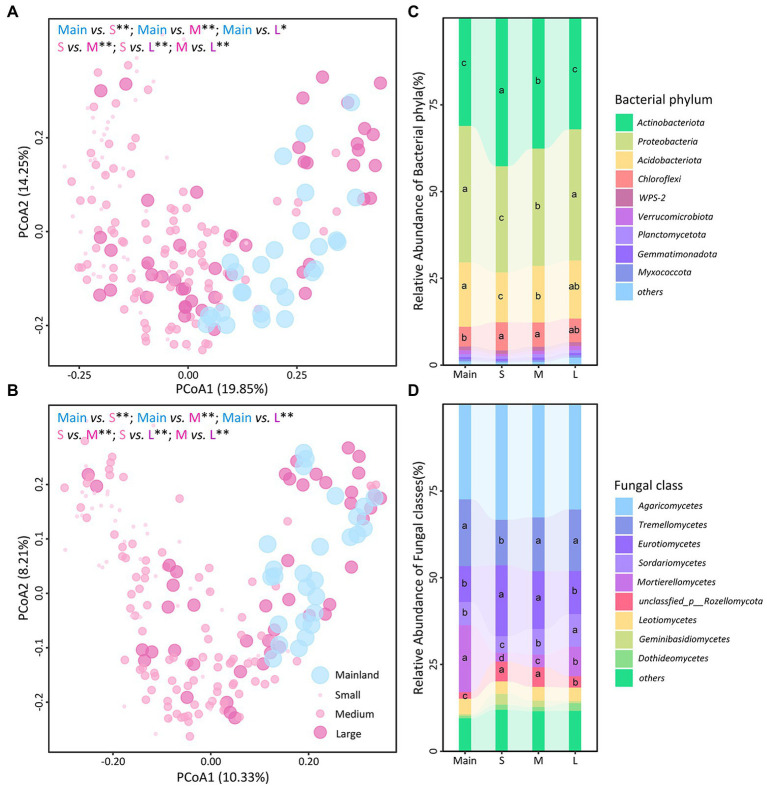
Soil bacterial (**A** and **C**) and fungal (**B** and **D**) community composition among the mainland (Main) and three island types (S: small islands; M: medium islands; L: large islands). Community composition was estimated by principal coordinate analysis (PCoA) based on Bray-Curtis dissimilarities **(A,B)**. Differences in community composition between mainland and each island type were assessed using permutational analysis of variance (*, *p* ≤ 0.05; **, *p* ≤ 0.01). Stacked bar plots indicate relative abundance (%) of bacterial phyla **(C)** and fungal classes **(D)** among the mainland and three island types, respectively. Different letters represent credibly different posterior distributions of relative abundance.

Permutational multivariate analysis of variance analysis showed that the soil bacterial and fungal communities differed among the three types of islands, as well as between each type of islands and the mainland (Figures 3A,B). The relative abundances of *Actinobacteriota* were higher on the islands than on the mainland but decreased from the small to large islands; the relative abundances of *Proteobacteriota* and *Acidobacteriota* were lower on the islands than on the mainland but increased from small to large islands (Figures 3C; Supplementary Tables S4–S6). Although the relative abundance of the dominant fungal class *Agaricomycetes* was constant, the relative abundances of other dominant fungal classes differed among the mainland and three types of islands (Figure 3D; Supplementary Tables S4–S6). In particular, the relative abundance of *Mortierellomycetes* was lower on the three types of islands than on the mainland and decreased from large to small islands. In contrast, the relative abundances of *Tremellomycetes* and *Eurotiomycetes* were higher on small islands than on the mainland or on large islands (Figure 3D; Supplementary Tables S4–S6). Results of microbial balances showed that the bacterial genera *Acidodthermus*, *Pseudonocardia*, and *Bacillus and* fungal genera *Sagenomella*, *Adisciso*, *Clavulina*, and *Trichoderma* were more predictive on three types of islands, while the bacterial genera *Roseiarcus*, *Kitasatospora*, and *Roseiarcus* and fungal genera *Mortierella* were more predictive on mainland (Supplementary Figure S4). Correlation analysis revealed that the relative abundances of dominant phylum/classes and microbial signatures were associated with most soil abiotic variables, especially for soil moisture and soil pH (Supplementary Figure S5; Table S7).

### Factors associated with microbial biomass, richness, and dissimilarity

**Figure 4:**
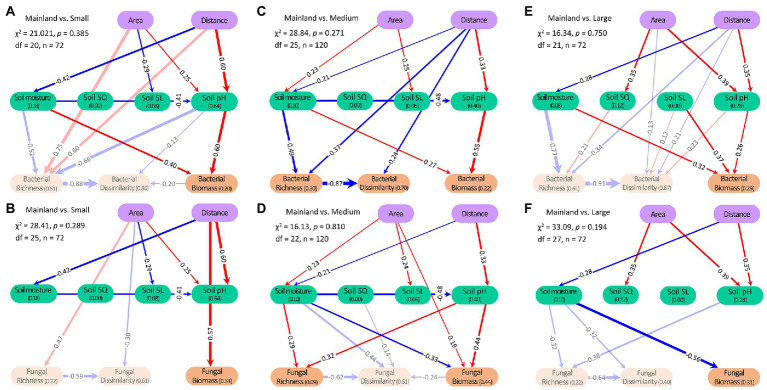
Structural equation model (SEM) for the response ratio of soil bacterial and fungal biomass, richness, and dissimilarity under small subdivision **(A,B)**, medium subdivision **(C,D)**, and large subdivision **(E,F)**. Soil SQ, soil substrate quantity; Soil SL, soil substrate quality. Arrows indicate significant (*p* ≤ 0.05) effects. Values associated with arrows represent standardized path coefficients; pathways without significant effects (*p* > 0.05) are not shown. Values associated with response variables in brackets indicate the proportion of variation (R^2^) explained by relationships with other variables. The variables with light shade in the SEM indicate they are not significantly altered (*p* > 0.05) by habitat fragmentation.

SEM showed that the factors associated with habitat subdivision-induced changes in microbial variables were different among the three types of islands. Compared with mainland, the higher bacterial biomass on the small-sized islands was mainly associated with changes in soil moisture and soil pH, while the higher fungal biomass on the small-sized islands was directly associated with distance-to-mainland (Figures 4A,B). The higher bacterial and fungal biomass on the medium islands than on the mainland were mainly associated with changes in soil moisture and soil pH (Figures 4C,D). Compared with mainland, the higher bacterial biomass on the large-sized islands was mainly associated with changes in soil nutrient quality, soil moisture, and soil pH, while the higher fungal biomass was associated with changes in soil moisture (Figures 4E,F). The higher soil bacterial richness on medium islands than on the mainland was mainly associated with soil moisture and direct effect of distance, while the higher fungal richness on the medium islands was mainly associated with soil moisture and pH (Figures 4C,D). The lower soil bacterial dissimilarity on medium islands than on the mainland was mainly associated with bacterial richness and direct effect of distance (Figure 4C).

**Figure 5:**
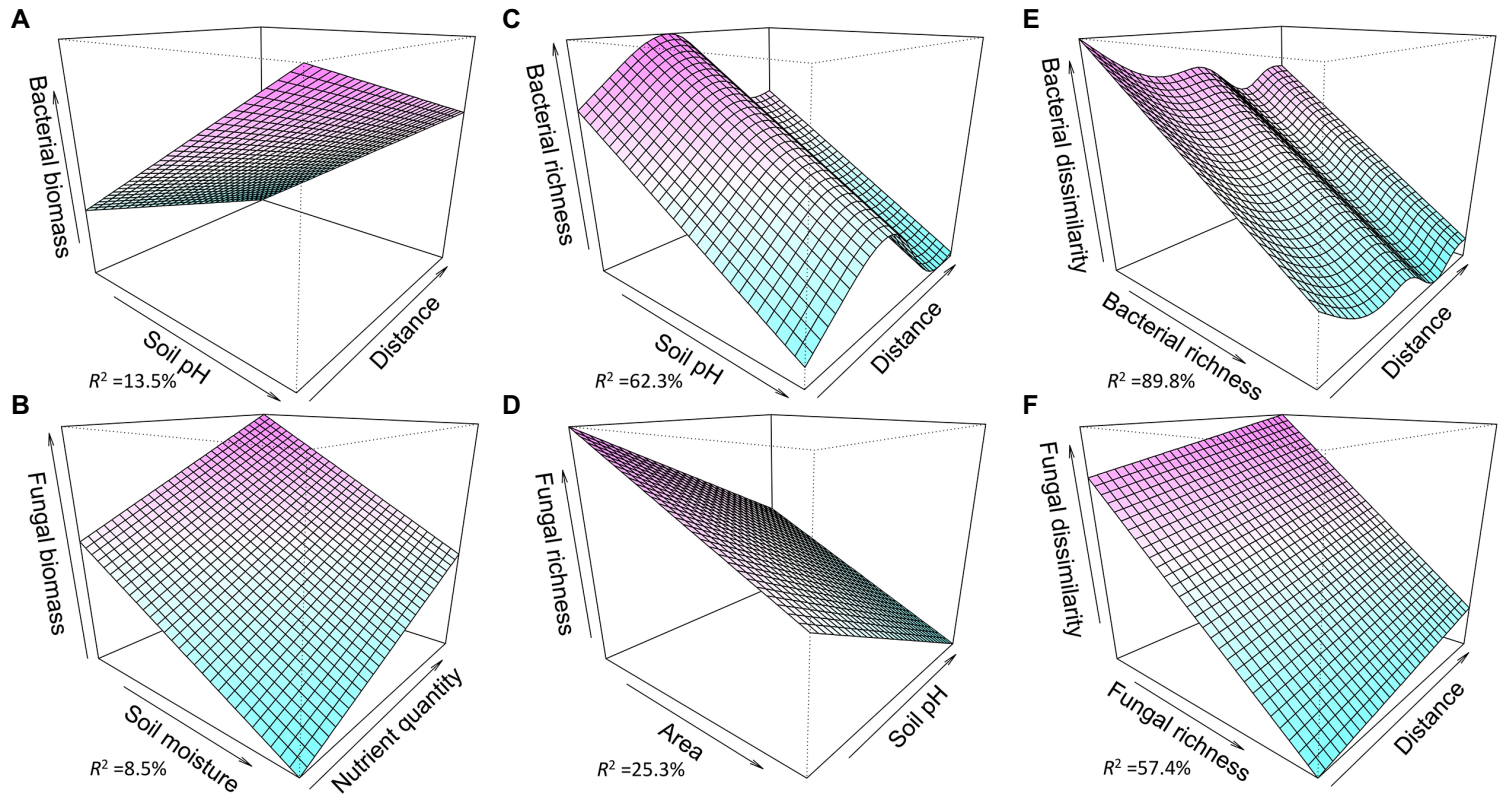
Fitted relationships between soil microbial variables (biomass, richness, and dissimilarity) and explanatory variables across the three island types based on generalized additive models. Bacterial biomass **(A)** and bacterial richness **(C)** were explained by distance and soil pH; fungal biomass **(B)** was explained by soil moisture and soil substrate quantity; fungal richness **(D)** was explained by island area and soil pH; bacterial dissimilarity **(E)** and fungal dissimilarity **(F)** were explained by their richness and distance to mainland. The detailed results of generalized additive models were showed in Supplementary Table S8.

Across the three island types, regression analyses showed that bacterial richness was not related to island area, but negatively related to distance to mainland, while fungal richness was negatively related to island area, but not related with distance to mainland (Supplementary Figure S6). SEM showed that the bacterial biomass was mainly associated with area-induced changes in soil moisture and soil nutrient quantity and distance-induced changes in soil pH (Supplementary Figures S7A,B). The fungal biomass was mainly associated with area-induced changes in soil moisture and soil nutrient quantity and distance-induced changes in soil pH (Supplementary Figures S7A,B). Soil bacterial richness was mainly associated with distance-induced changes in soil nutrient quantity and soil pH, and direct effect of distance (Supplementary Figures S7A,B). The fungal richness was mainly associated with distance-induced changes in soil pH (Supplementary Figures S7A,B). Soil bacterial or fungal dissimilarity was mainly associated with their richness (Supplementary Figure S7). Further, GAM analysis further showed that soil bacterial biomass was negatively associated with distance to mainland and was positively associated with soil pH; soil fungal biomass was negatively associated with soil moisture and was positively associated with soil substrate quantity (Figures 5A,B; Supplementary Figure S8, Table S8). Soil bacterial richness had unimodal relationship with distance to mainland and had negative linear relationship with soil pH; soil fungal richness had negative relationships with island area and soil pH (Figures 5C,D; Supplementary Figure S8, Table S8). Soil bacterial dissimilarity had negative relationship with soil bacterial richness and distance to mainland; soil fungal dissimilarity had negative relationship with soil fungal richness and had positive relationship with distance to mainland (Figures 5E,F; Supplementary Figure S8, Table S8).

## Discussion

We found that both soil bacterial and fungal biomass were higher on islands than that on mainland, but the degree of changes depended on types of subdivision and their associated habitat heterogeneity, which consistent with our first hypothesis. Across three types of islands, area- or distance-induced increase in soil pH consistently enhanced bacterial biomass. The strong and positive associations between soil microbial biomass and soil pH were well reported at different ecosystems due to the narrow pH niche for soil microbial communities (Rousk et al., 2009; Chen et al., 2015). The greatest change in bacterial biomass of large subdivision was attributed to combined effects of soil pH and soil nutrient quality which indicated that higher habitat heterogeneity in larger island may play an important role in bacterial biomass (Li et al., 2020b). In contrast, soil fungal biomass was promoted by distance-induced decrease in soil moisture because the higher soil moisture in the subtropical forests might inhibit the growth of soil fungi (Cruz-Paredes et al., 2021). In addition, habitat subdivision consistently decreased B:F ratio on each type of islands, suggesting that soils on mainland are dominated by bacterial-based energy channels while soils on each of the three types of islands are dominated by fungal-based energy channels. The shift from bacterial-based to fungal-based energy channels resulting from habitat subdivision could result in decreases in soil nutrient losses and CO_2_ release, which in turn could cause the fragmented islands to be more resistance to climate changes (de Vries et al., 2013; Chen et al., 2016).

In contrast to our first hypothesis, only on the medium-sized islands, soil bacterial and fungal richness were higher than that on the mainland, which was somewhat consistent with the intermediate disturbance hypothesis (Connell, 1978; Huston, 1979). That is to say, changes in bacterial and fungal richness were mainly derived from extrinsic process and associated intrinsic competition (Raynaud and Nunan, 2014). For example, the increase in bacterial richness was attributed to that lower moisture could promote competition ability of subdominant species (Carson et al., 2010); the increase in fungal richness was attributed to that higher soil pH could benefit ectomycorrhizal fungi growth and reproduction (Wardle and Lindahl, 2014). The lack differences in microbial richness between mainland and small or large islands could be due to the offset effects among various external environment (Raynaud and Nunan, 2014). The strong differences in microbial biomass between mainland and islands were highly associated changes in habitat heterogeneity resulting from changes in island geometrical properties (Yu et al., 2012). Normally, microbial local interactions are likely to be obscured by relatively large samples which encompass high environmental heterogeneity (Raynaud and Nunan, 2014). The consistent and positive relationships between microbial richness and dissimilarity in any subdivision cases showed that the subdivision process may encounter strong microbial interactions. Bacterial dissimilarity was lowest on medium-sized islands could perhaps be due to the reduced fitness of the original dominant, mainland bacterial taxa on medium-sized islands and to the increased fitness of previously subdominant competitors that were favored by medium-sized islands (Chesson, 2000; Mouquet and Loreau, 2002). The lower sensitive of soil fungal dissimilarity to habitat subdivision than bacterial fungal similarity could be due to the fact that soil fungi have mycelial networks and are more resistant than bacteria to environmental disturbances (Rousk et al., 2010; Wang et al., 2020). The strong negative relationships between microbial richness and dissimilarity without reference to island size suggests that these fragmented habitats may still be experiencing the replacement of the previously most competitive taxa by other competitors (Chesson, 2000; Mouquet and Loreau, 2002).

Our permutational multivariate analysis of variance analysis showed that both soil bacterial and fungal community composition were significantly changed by long-term habitat subdivision. From small to large islands, the relative abundance decreased for the dominant bacterial phylum *Actinobacteriota* but increased for the dominant bacterial phylum *Proteobacteriota*, although both phyla were previously characterized as copiotrophic based on classical life strategy theory (Fierer et al., 2007). The opposite relationships of the relative abundance of *Actinobacteriota* and *Proteobacteriota* with soil nutrients (SOC and TN) on the subtropical land-bridge islands indicates that these taxa still had different preferences for substrates and nutrients, although previous studies showed that the relative abundance of both phyla were positively correlated with soil nutrients (Lauber et al., 2008; Fierer et al., 2011; Laliberté et al., 2017). We also found that the relative abundance of most bacterial phyla and fungal classes were related to soil moisture and soil pH on the subtropical land-bridge islands, which is consistent with previous studies (Rousk et al., 2010; Chen et al., 2016, 2019; Wu et al., 2020). Our research showed that changes in the relationships between the relative abundance of several fungal classes after fragmentation with soil properties (such as the negative relationships between *Agaricomycetes* and soil pH and soil moisture, and the positive relationships between *Eurotiomycetes* and soil pH and total N) were also consistent with other studies (Hartmann et al., 2017; Li et al., 2020a). In addition, based on the reduction in the relative abundance of *Mortierellomycetes*, we infer that fungi are more closely related than bacteria to vegetation (Li et al., 2020a). Differences in microbial signatures between mainland and island also derived from soil properties, especially soil moisture and soil pH (Supplementary Figures S4, S5). For example, bacterial genus *Roseiarcus* and *Kitasatospora* were preferred on mainland due to their negative relationships with soil pH (Kämpfer, 2006; Rezaei et al., 2011; Kulichevskaya et al., 2014; Riahi et al., 2022), and fungal genus *Mortierella* was preferred on mainland due to its close interaction with plant roots (Toju and Sato, 2018). Overall, we found that habitat fragmentation greatly altered the composition of soil bacterial and fungal communities for each island size, and that the soil and plant properties associated with the habitat fragment-induced changes in community composition differed for bacteria and fungi.

Our ordinary least squares regression and structural equation model found only the relationship between bacterial richness and distance to mainland was consistent with species-distance relationships, while microbial richness was completely inconsistent with species-area relationships (MacArthur and Wilson, 2016). In contrast, both microbial biomass and diversity of soil bacteria or fungi were mainly associated with soil abiotic variables. Soil pH was still strongly associated with soil microbial biomass and richness (Rousk et al., 2009), although there were anti-directional responses in bacterial biomass and richness to soil pH might be due to the disproportionate contributions of dominant taxa to biomass than subdominant taxa (Bastida et al., 2021). We found soil fungal biomass decreased with increased soil moisture and increased with soil substrate quantity. The increase of soil carbon and nutrient content could provide more substrates for fungi growth (Bastida et al., 2021), however, negative effects of soil moisture on soil fungal biomass indicated that higher water content may inhibit their mobility to capture soil nutrients (Manzoni et al., 2012). Partly consistent with another land-bridge study (Li et al., 2020b), our results showed the island area had less effects on soil bacterial and fungal richness than distance to the mainland across the three types of islands, and this was especially the case for bacterial richness. Generalized additive model analysis showed that there were unimodal relationships between distance to mainland and bacterial richness across the three types of islands. Based on the coexistence theory of metacommunities (Mouquet and Loreau, 2002; Loreau et al., 2003) and as previously noted the intermediate disturbance hypothesis (Connell, 1978; Huston, 1979), the highest biodiversity may occur at a medium dispersal level because both species homogenization caused by high dispersal and competitive exclusion caused by low dispersal would reduce diversity. Our results also showed that soil bacterial or fungal dissimilarity were mainly associated with their richness and distance to mainland across the three types of islands. The robust negative relationships between microbial richness and dissimilarity across the three types of islands confirmed that species coexistence was inhibited when the community became more dissimilar (Mouquet and Loreau, 2002). Furthermore, we found that the variables associated with soil fungal richness or dissimilarity had less explanatory power than the variables associated with soil bacteria richness perhaps because some functional groups of soil fungi (e.g., ectomycorrhizal fungi) are more closely related than soil bacteria to the changes in vegetation composition (Peay et al., 2007). Across the three types of islands, we found that the richness of soil microorganisms was mainly associated with island area and distance to the mainland while soil microbial dissimilarity was mainly associated with microbial richness and distance to the mainland.

## Conclusion

Using 61 subtropical land-bridge islands with a 50-year history of subdivision and 9 adjacent mainland sites as a model system, we highlight several findings: Firstly, soil bacterial and fungal biomass in different-sized subdivision were higher than that in mainland, while increased the soil bacterial and fungal richness only on the medium-sized islands. These changes were due to subdivision-specified habitat heterogeneity, especial for soil moisture and soil pH. Secondly, soil microbial dissimilarities were robustly and negatively associated with microbial richness suggests that these fragmented habitats may still be experiencing the replacement of the previously most competitive taxa by other competitors. Finally, soil microbial distributions are not consistent with species-area relationships, but determined by habitat heterogeneity and tradeoff between competition and dispersal.

## Data availability statement

The datasets presented in this study can be found in online repositories. The names of the repository/repositories and accession number(s) can be found below: https://www.ncbi.nlm.nih.gov/, PRJNA898793. The bacterial and fungal ASV table and relevant environmental variables is available on GitHub at https://github.com/DimaChen-ctgu/Island-2020.

## Author contributions

All authors contributed to the article and approved the submitted version, and due care has been taken to ensure the integrity of the work. The accompanying manuscript constitutes original unpublished work and is not under consideration for publication elsewhere.

## Funding

This study was supported by the National Natural Science Foundation of China (42177272), and the Youth Innovation Promotion Association of the Chinese Academy of Sciences (2015061).

## Conflict of interest

The authors declare that the research was conducted in the absence of any commercial or financial relationships that could be construed as a potential conflict of interest.

## Publisher’s note

All claims expressed in this article are solely those of the authors and do not necessarily represent those of their affiliated organizations, or those of the publisher, the editors and the reviewers. Any product that may be evaluated in this article, or claim that may be made by its manufacturer, is not guaranteed or endorsed by the publisher.

## Supplementary material

The Supplementary material for this article can be found online at: https://www.frontiersin.org/articles/10.3389/fmicb.2022.1063340/full#supplementary-material
