# Supplementary material for: Fifty-year habitat subdivision enhances soil microbial biomass and diversity across subtropical land-bridge islands
Source: Front Microbiol. 2022 Dec 9;13:1063340. doi: 10.3389/fmicb.2022.1063340 (PMC9780280; doi:10.3389/fmicb.2022.1063340)
Supplement: Supplementary file 1 [file Data_Sheet_1.docx]

**Supporting Information**

**Table S1** Summary statistics of the posterior distributions of soil abiotic variables among the mainland and three land-area types (S, small islands; M, medium islands; L, large islands) using the MCMC method.

| **Variable** | **Comparison** | **mean** | **sd** | **2.50%** | **50%** | **97.50%** | **Rhat** | **n.eff** |
| --- | --- | --- | --- | --- | --- | --- | --- | --- |
| Soil moisture | Main *vs.* S | 0.139 | 0.015 | 0.109 | 0.139 | 0.168 | 1 | 40000 |
|  | Main *vs.* M | 0.094 | 0.014 | 0.067 | 0.094 | 0.121 | 1 | 40311 |
|  | S *vs.* M | -0.045 | 0.010 | -0.065 | -0.045 | -0.024 | 1 | 40448 |
|  | Main *vs.* L | 0.041 | 0.018 | 0.006 | 0.041 | 0.076 | 1 | 40105 |
|  | S *vs.* L | -0.097 | 0.015 | -0.127 | -0.097 | -0.067 | 1 | 40000 |
|  | M *vs.* L | -0.052 | 0.014 | -0.080 | -0.052 | -0.024 | 1 | 40000 |
| Soil pH | Main *vs.* S | -0.240 | 0.053 | -0.343 | -0.240 | -0.136 | 1 | 40000 |
|  | Main *vs.* M | -0.193 | 0.041 | -0.275 | -0.193 | -0.113 | 1 | 40000 |
|  | S *vs.* M | 0.046 | 0.043 | -0.039 | 0.046 | 0.132 | 1 | 39529 |
|  | Main *vs.* L | -0.250 | 0.057 | -0.361 | -0.249 | -0.140 | 1 | 39732 |
|  | S *vs.* L | -0.010 | 0.058 | -0.124 | -0.010 | 0.105 | 1 | 40000 |
|  | M *vs.* L | -0.056 | 0.048 | -0.150 | -0.056 | 0.038 | 1 | 38693 |
| SOC | Main *vs.* S | 0.117 | 0.280 | -0.434 | 0.118 | 0.669 | 1 | 39443 |
|  | Main *vs.* M | -0.317 | 0.214 | -0.739 | -0.317 | 0.104 | 1 | 39687 |
|  | S *vs.* M | -0.434 | 0.251 | -0.925 | -0.435 | 0.062 | 1 | 38477 |
|  | Main *vs.* L | 0.289 | 0.256 | -0.216 | 0.290 | 0.793 | 1 | 40313 |
|  | S *vs.* L | 0.172 | 0.288 | -0.393 | 0.173 | 0.743 | 1 | 38315 |
|  | M *vs.* L | 0.606 | 0.226 | 0.162 | 0.606 | 1.050 | 1 | 40968 |
| TSN | Main *vs.* S | 0.037 | 0.017 | 0.003 | 0.037 | 0.070 | 1 | 40690 |
|  | Main *vs.* M | 0.021 | 0.013 | -0.006 | 0.021 | 0.047 | 1 | 40330 |
|  | S *vs.* M | -0.016 | 0.013 | -0.042 | -0.016 | 0.010 | 1 | 39679 |
|  | Main *vs.* L | 0.020 | 0.017 | -0.014 | 0.020 | 0.054 | 1 | 40000 |
|  | S *vs.* L | -0.017 | 0.017 | -0.050 | -0.017 | 0.016 | 1 | 40000 |
|  | M *vs.* L | -0.001 | 0.014 | -0.028 | -0.001 | 0.026 | 1 | 40000 |
| TSP | Main *vs.* S | 0.002 | 0.002 | -0.002 | 0.002 | 0.006 | 1 | 40000 |
|  | Main *vs.* M | 0.002 | 0.002 | -0.002 | 0.002 | 0.005 | 1 | 40000 |
|  | S *vs.* M | 0.000 | 0.001 | -0.002 | 0.000 | 0.002 | 1 | 40427 |
|  | Main *vs.* L | -0.003 | 0.003 | -0.009 | -0.003 | 0.003 | 1 | 40650 |
|  | S *vs.* L | -0.005 | 0.003 | -0.011 | -0.005 | 0.000 | 1 | 40507 |
|  | M *vs.* L | -0.005 | 0.003 | -0.010 | -0.005 | 0.000 | 1 | 40289 |
| Soil C:N | Main *vs.* S | -1.413 | 0.641 | -2.671 | -1.411 | -0.164 | 1 | 39212 |
|  | Main *vs.* M | -2.296 | 0.564 | -3.406 | -2.295 | -1.182 | 1 | 39609 |
|  | S *vs.* M | -0.883 | 0.520 | -1.899 | -0.885 | 0.141 | 1 | 38254 |
|  | Main *vs.* L | 0.182 | 0.641 | -1.078 | 0.179 | 1.437 | 1 | 40000 |
|  | S *vs.* L | 1.595 | 0.600 | 0.424 | 1.595 | 2.775 | 1 | 40250 |
|  | M *vs.* L | 2.477 | 0.519 | 1.461 | 2.477 | 3.492 | 1 | 39413 |
| Soil C:P | Main *vs.* S | 1.901 | 10.233 | -18.241 | 1.901 | 22.071 | 1 | 40211 |
|  | Main *vs.* M | -19.802 | 9.261 | -38.233 | -19.770 | -1.802 | 1 | 40000 |
|  | S *vs.* M | -21.704 | 8.807 | -38.929 | -21.663 | -4.478 | 1 | 42557 |
|  | Main *vs.* L | 17.829 | 10.241 | -2.072 | 17.899 | 37.910 | 1 | 40047 |
|  | S *vs.* L | 15.928 | 9.828 | -3.386 | 15.963 | 35.174 | 1 | 41029 |
|  | M *vs.* L | 37.632 | 8.786 | 20.371 | 37.614 | 55.101 | 1 | 40000 |
| Soil N:P | Main *vs.* S | 1.107 | 0.518 | 0.092 | 1.105 | 2.125 | 1 | 39701 |
|  | Main *vs.* M | 0.218 | 0.459 | -0.693 | 0.220 | 1.125 | 1 | 39644 |
|  | S *vs.* M | -0.888 | 0.427 | -1.728 | -0.885 | -0.052 | 1 | 39859 |
|  | Main *vs.* L | 1.264 | 0.522 | 0.239 | 1.262 | 2.286 | 1 | 38976 |
|  | S *vs.* L | 0.157 | 0.493 | -0.808 | 0.158 | 1.123 | 1 | 39604 |
|  | M *vs.* L | 1.046 | 0.428 | 0.206 | 1.046 | 1.885 | 1 | 38663 |

**Table S2** Summary statistics of the island attributes among the mainland and three land-area types (S, small islands; M, medium islands; L, large islands) using the MCMC method.

| **Variable** | **Comparison** | **mean** | **sd** | **2.50%** | **50%** | **97.50%** | **Rhat** | **n.eff** |
| --- | --- | --- | --- | --- | --- | --- | --- | --- |
| Area | S *vs.* M | -0.253 | 0.017 | -0.287 | -0.253 | -0.219 | 1 | 40267 |
|  | S *vs.* L | -1.205 | 0.076 | -1.353 | -1.205 | -1.055 | 1 | 40000 |
|  | M *vs.* L | -0.952 | 0.077 | -1.101 | -0.952 | -0.801 | 1 | 39703 |
| Distance-to-mainland | S *vs.* M | 0.232 | 0.171 | -0.106 | 0.232 | 0.567 | 1 | 40000 |
|  | S *vs.* L | 0.481 | 0.201 | 0.088 | 0.480 | 0.875 | 1 | 39710 |
|  | M *vs.* L | 0.249 | 0.169 | -0.082 | 0.250 | 0.582 | 1 | 38854 |
| Nearest distance | S *vs.* M | -0.170 | 0.085 | -0.337 | -0.171 | -0.003 | 1 | 40000 |
|  | S *vs.* L | -0.860 | 0.128 | -1.112 | -0.860 | -0.612 | 1 | 38844 |
|  | M *vs.* L | -0.690 | 0.112 | -0.909 | -0.690 | -0.470 | 1 | 40396 |
| Perimeter | S *vs.* M | -0.763 | 0.072 | -0.905 | -0.763 | -0.622 | 1 | 40000 |
|  | S *vs.* L | -2.511 | 0.173 | -2.853 | -2.510 | -2.169 | 1 | 40556 |
|  | M *vs.* L | -1.748 | 0.169 | -2.079 | -1.748 | -1.414 | 1 | 40000 |
| Perimeter: area ratio | S *vs.* M | 0.018 | 0.002 | 0.014 | 0.018 | 0.022 | 1 | 40000 |
|  | S *vs.* L | 0.042 | 0.002 | 0.038 | 0.042 | 0.046 | 1 | 40071 |
|  | M *vs.* L | 0.025 | 0.002 | 0.021 | 0.025 | 0.028 | 1 | 40285 |
| Shape index | S *vs.* M | -36.034 | 6.728 | -49.208 | -36.045 | -22.886 | 1 | 40000 |
|  | S *vs.* L | -151.146 | 33.287 | -214.799 | -151.967 | -83.820 | 1 | 40401 |
|  | M *vs.* L | -115.111 | 33.475 | -178.713 | -115.864 | -47.352 | 1 | 40321 |

**Table S3** Summary statistics of the posterior distributions of the differences in microbial biomass, richness, and dissimilarity among the mainland and three land-area types (S, small islands; M, medium islands; L, large islands) using the MCMC method.

| **Variable** | **Comparison** | **mean** | **sd** | **2.50%** | **50%** | **97.50%** | **Rhat** | **n.eff** |
| --- | --- | --- | --- | --- | --- | --- | --- | --- |
| Bacterial biomass | Main vs S | -2.650 | 1.037 | -4.687 | -2.651 | -0.600 | 1 | 40000 |
|  | Main vs M | -2.985 | 0.888 | -4.729 | -2.988 | -1.235 | 1 | 39657 |
|  | S vs M | -0.334 | 0.923 | -2.138 | -0.334 | 1.490 | 1 | 39414 |
|  | Main vs L | -6.500 | 1.187 | -8.822 | -6.497 | -4.168 | 1 | 39802 |
|  | S vs L | -3.849 | 1.221 | -6.244 | -3.847 | -1.459 | 1 | 39577 |
|  | M vs L | -3.515 | 1.090 | -5.657 | -3.518 | -1.357 | 1 | 39761 |
| Fungal biomass | Main vs S | -0.922 | 0.123 | -1.162 | -0.923 | -0.680 | 1 | 40010 |
|  | Main vs M | -0.794 | 0.066 | -0.925 | -0.794 | -0.663 | 1 | 40568 |
|  | S vs M | 0.128 | 0.121 | -0.110 | 0.129 | 0.366 | 1 | 39788 |
|  | Main vs L | -0.686 | 0.098 | -0.879 | -0.686 | -0.492 | 1 | 40542 |
|  | S vs L | 0.236 | 0.140 | -0.042 | 0.236 | 0.510 | 1 | 39872 |
|  | M vs L | 0.108 | 0.096 | -0.082 | 0.107 | 0.296 | 1 | 41036 |
| B:F ratio | Main vs S | 8.573 | 0.902 | 6.808 | 8.571 | 10.367 | 1 | 39630 |
|  | Main vs M | 7.892 | 0.848 | 6.232 | 7.889 | 9.567 | 1 | 39717 |
|  | S vs M | -0.680 | 0.538 | -1.738 | -0.677 | 0.370 | 1 | 40000 |
|  | Main vs L | 3.975 | 1.189 | 1.653 | 3.977 | 6.301 | 1 | 40538 |
|  | S vs L | -4.598 | 0.991 | -6.558 | -4.599 | -2.642 | 1 | 39219 |
|  | M vs L | -3.917 | 0.948 | -5.783 | -3.918 | -2.049 | 1 | 39297 |
| Bacterial richness | Main vs S | -0.136 | 20.298 | -40.119 | -0.165 | 39.746 | 1 | 40165 |
|  | Main vs M | -50.094 | 16.697 | -83.400 | -49.843 | -17.948 | 1 | 40419 |
|  | S vs M | -49.957 | 18.942 | -87.532 | -49.756 | -13.166 | 1 | 40095 |
|  | Main vs L | 24.733 | 25.747 | -25.080 | 24.482 | 76.302 | 1 | 40607 |
|  | S vs L | 24.869 | 27.154 | -28.047 | 24.803 | 79.076 | 1 | 40372 |
|  | M vs L | 74.827 | 24.761 | 27.066 | 74.551 | 124.462 | 1 | 39029 |
| Fungal richness | Main vs S | -9.400 | 7.475 | -24.190 | -9.359 | 5.193 | 1 | 40000 |
|  | Main vs M | -11.478 | 5.672 | -22.643 | -11.464 | -0.338 | 1 | 40000 |
|  | S vs M | -2.078 | 6.696 | -15.328 | -2.057 | 11.012 | 1 | 39992 |
|  | Main vs L | 0.534 | 7.536 | -14.132 | 0.537 | 15.346 | 1 | 40383 |
|  | S vs L | 9.935 | 8.325 | -6.354 | 9.941 | 26.220 | 1 | 40000 |
|  | M vs L | 12.012 | 6.800 | -1.316 | 11.994 | 25.603 | 1 | 39218 |
| Bacterial dissimilarity | Main vs S | 0.035 | 0.021 | -0.006 | 0.035 | 0.077 | 1 | 40327 |
|  | Main vs M | 0.061 | 0.020 | 0.021 | 0.061 | 0.100 | 1 | 40022 |
|  | S vs M | 0.025 | 0.009 | 0.008 | 0.025 | 0.043 | 1 | 40480 |
|  | Main vs L | 0.026 | 0.023 | -0.020 | 0.026 | 0.071 | 1 | 40452 |
|  | S vs L | -0.010 | 0.014 | -0.038 | -0.010 | 0.019 | 1 | 41150 |
|  | M vs L | -0.035 | 0.013 | -0.060 | -0.035 | -0.010 | 1 | 41305 |
| Fungal dissimilarity | Main vs S | 0.024 | 0.010 | 0.004 | 0.024 | 0.044 | 1 | 41097 |
|  | Main vs M | 0.035 | 0.008 | 0.018 | 0.035 | 0.051 | 1 | 38499 |
|  | S vs M | 0.011 | 0.008 | -0.005 | 0.011 | 0.026 | 1 | 40000 |
|  | Main vs L | 0.019 | 0.010 | 0.000 | 0.019 | 0.038 | 1 | 39973 |
|  | S vs L | -0.005 | 0.009 | -0.023 | -0.005 | 0.013 | 1 | 40359 |
|  | M vs L | -0.015 | 0.007 | -0.029 | -0.015 | -0.001 | 1 | 39461 |

**Table S4** Mean relative abundance of bacterial phyla and fungal classes among the mainland and three land-area types. Different lowercase letters indicate credibly different posterior distributions (95% HDI of the posterior difference between each two groups falls above or below zero).

|  | **Phyla or Classes** | **Mainland** | **Small** | **Medium** | **Large** |
| --- | --- | --- | --- | --- | --- |
| Bacteria | *Actinobacteriota* | 0.311^c^ | 0.427^a^ | 0.376^b^ | 0.321^c^ |
|  | *Proteobacteria* | 0.393^a^ | 0.306^c^ | 0.338^b^ | 0.378^a^ |
|  | *Acidobacteriota* | 0.185^a^ | 0.144^c^ | 0.163^b^ | 0.167^ab^ |
|  | *Chloroflexi* | 0.057^b^ | 0.081^a^ | 0.07^a^ | 0.068^ab^ |
|  | *WPS-2* | 0.011^a^ | 0.01^a^ | 0.012^a^ | 0.011^a^ |
|  | *Verrucomicrobiota* | 0.011^ab^ | 0.007^c^ | 0.009^b^ | 0.012^a^ |
|  | *Planctomycetota* | 0.008^ab^ | 0.007^b^ | 0.01^a^ | 0.008^b^ |
|  | *Gemmatimonadota* | 0.007^a^ | 0.007^a^ | 0.007^a^ | 0.007^a^ |
|  | *Myxococcota* | 0.006^ab^ | 0.004^c^ | 0.005^b^ | 0.007^a^ |
| Fungi | *Agaricomycetes* | 0.275^a^ | 0.333^a^ | 0.326^a^ | 0.304^a^ |
|  | *Tremellomycetes* | 0.192^a^ | 0.131^b^ | 0.155^a^ | 0.177^a^ |
|  | *Eurotiomycetes* | 0.103^b^ | 0.205^a^ | 0.167^a^ | 0.124^b^ |
|  | *Sordariomycetes* | 0.067^b^ | 0.048^c^ | 0.074^b^ | 0.094^a^ |
|  | *Mortierellomycetes* | 0.193^a^ | 0.026^d^ | 0.036^c^ | 0.085^b^ |
|  | *unclassfied_Rozellomycota* | 0.019^c^ | 0.057^a^ | 0.057^a^ | 0.033^b^ |
|  | *Leotiomycetes* | 0.044^a^ | 0.036^a^ | 0.04^a^ | 0.039^a^ |
|  | *Geminibasidiomycetes* | 0.005^b^ | 0.031^a^ | 0.018^a^ | 0.006^b^ |
|  | *Dothideomycetes* | 0.007^b^ | 0.015^a^ | 0.012^a^ | 0.022^a^ |

**Table S5** Summary statistics of the posterior distributions of the differences in the relative abundance of bacterial phyla among the mainland and three land-area types (S, small islands; M, medium islands; L, large islands) using the MCMC method.

| **Variable** | **Comparison** | **mean** | **sd** | **2.50%** | **50%** | **97.50%** | **Rhat** | **n.eff** |
| --- | --- | --- | --- | --- | --- | --- | --- | --- |
| *Actinobacteriota* | Main *vs.* S | -0.116 | 0.015 | -0.145 | -0.116 | -0.087 | 1 | 38720 |
|  | Main *vs.* M | -0.065 | 0.013 | -0.090 | -0.065 | -0.040 | 1 | 40000 |
|  | S *vs.* M | 0.051 | 0.014 | 0.023 | 0.051 | 0.079 | 1 | 39012 |
|  | Main *vs.* L | -0.010 | 0.017 | -0.042 | -0.010 | 0.023 | 1 | 40000 |
|  | S *vs.* L | 0.106 | 0.018 | 0.071 | 0.106 | 0.141 | 1 | 38120 |
|  | M *vs.* L | 0.055 | 0.016 | 0.024 | 0.055 | 0.087 | 1 | 39431 |
| *Proteobacteria* | Main *vs.* S | 0.087 | 0.012 | 0.063 | 0.087 | 0.111 | 1 | 40402 |
|  | Main *vs.* M | 0.055 | 0.012 | 0.032 | 0.055 | 0.078 | 1 | 40000 |
|  | S *vs.* M | -0.032 | 0.010 | -0.052 | -0.032 | -0.014 | 1 | 40434 |
|  | Main *vs.* L | 0.015 | 0.016 | -0.016 | 0.015 | 0.046 | 1 | 40362 |
|  | S *vs.* L | -0.072 | 0.015 | -0.101 | -0.072 | -0.044 | 1 | 40000 |
|  | M *vs.* L | -0.040 | 0.014 | -0.067 | -0.040 | -0.012 | 1 | 40000 |
| *Acidobacteriota* | Main *vs.* S | 0.041 | 0.011 | 0.019 | 0.041 | 0.063 | 1 | 38226 |
|  | Main *vs.* M | 0.022 | 0.011 | 0.001 | 0.022 | 0.043 | 1 | 39883 |
|  | S *vs.* M | -0.019 | 0.008 | -0.036 | -0.019 | -0.003 | 1 | 39785 |
|  | Main *vs.* L | 0.018 | 0.012 | -0.005 | 0.018 | 0.041 | 1 | 39465 |
|  | S *vs.* L | -0.024 | 0.010 | -0.043 | -0.024 | -0.004 | 1 | 38495 |
|  | M *vs.* L | -0.004 | 0.009 | -0.022 | -0.004 | 0.013 | 1 | 40327 |
| *Chloroflexi* | Main *vs.* S | -0.024 | 0.008 | -0.039 | -0.024 | -0.008 | 1 | 39685 |
|  | Main *vs.* M | -0.012 | 0.006 | -0.024 | -0.012 | -0.001 | 1 | 39705 |
|  | S *vs.* M | 0.011 | 0.007 | -0.003 | 0.011 | 0.026 | 1 | 39556 |
|  | Main *vs.* L | -0.011 | 0.007 | -0.024 | -0.011 | 0.002 | 1 | 39695 |
|  | S *vs.* L | 0.013 | 0.008 | -0.003 | 0.013 | 0.029 | 1 | 40000 |
|  | M *vs.* L | 0.002 | 0.006 | -0.010 | 0.002 | 0.013 | 1 | 40277 |
| *WPS-2* | Main *vs.* S | 0.001 | 0.001 | -0.002 | 0.001 | 0.003 | 1 | 40695 |
|  | Main *vs.* M | -0.001 | 0.001 | -0.003 | -0.001 | 0.001 | 1 | 39713 |
|  | S *vs.* M | -0.002 | 0.001 | -0.004 | -0.002 | 0.000 | 1 | 40008 |
|  | Main *vs.* L | 0.000 | 0.001 | -0.002 | 0.000 | 0.003 | 1 | 39361 |
|  | S *vs.* L | -0.001 | 0.001 | -0.003 | -0.001 | 0.002 | 1 | 40000 |
|  | M *vs.* L | 0.001 | 0.001 | -0.001 | 0.001 | 0.003 | 1 | 39791 |

**Table S6** Summary statistics of the posterior distributions of the differences in the relative abundance of fungal classes among the mainland and three land-area types (S, small islands; M, medium islands; L, large islands) using the MCMC method.

| **Fungal class** | **Comparison** | **mean** | **sd** | **2.50%** | **50%** | **97.50%** | **Rhat** | **n.eff** |
| --- | --- | --- | --- | --- | --- | --- | --- | --- |
| *Agaricomycetes* | Main *vs.* S | -0.059 | 0.044 | -0.146 | -0.059 | 0.027 | 1 | 41767 |
|  | Main *vs.* M | -0.052 | 0.041 | -0.134 | -0.052 | 0.029 | 1 | 41344 |
|  | S *vs.* M | 0.007 | 0.028 | -0.049 | 0.007 | 0.062 | 1 | 39135 |
|  | Main *vs.* L | -0.029 | 0.047 | -0.122 | -0.029 | 0.063 | 1 | 40417 |
|  | S *vs.* L | 0.030 | 0.036 | -0.042 | 0.030 | 0.102 | 1 | 41013 |
|  | M *vs.* L | 0.023 | 0.033 | -0.042 | 0.023 | 0.088 | 1 | 40000 |
| *Tremellomycetes* | Main *vs.* S | 0.061 | 0.023 | 0.016 | 0.061 | 0.107 | 1 | 40317 |
|  | Main *vs.* M | 0.037 | 0.021 | -0.003 | 0.037 | 0.078 | 1 | 39665 |
|  | S *vs.* M | -0.024 | 0.019 | -0.061 | -0.024 | 0.014 | 1 | 40568 |
|  | Main *vs.* L | 0.016 | 0.025 | -0.033 | 0.016 | 0.065 | 1 | 39458 |
|  | S *vs.* L | -0.046 | 0.024 | -0.093 | -0.046 | 0.002 | 1 | 40488 |
|  | M *vs.* L | -0.022 | 0.021 | -0.063 | -0.022 | 0.020 | 1 | 39418 |
| *Eurotiomycetes* | Main *vs.* S | -0.101 | 0.023 | -0.147 | -0.101 | -0.056 | 1 | 39657 |
|  | Main *vs.* M | -0.063 | 0.018 | -0.098 | -0.063 | -0.029 | 1 | 40713 |
|  | S *vs.* M | 0.038 | 0.021 | -0.003 | 0.038 | 0.078 | 1 | 40000 |
|  | Main *vs.* L | -0.021 | 0.019 | -0.059 | -0.021 | 0.018 | 1 | 40819 |
|  | S *vs.* L | 0.080 | 0.022 | 0.037 | 0.080 | 0.124 | 1 | 40000 |
|  | M *vs.* L | 0.042 | 0.016 | 0.010 | 0.042 | 0.074 | 1 | 40662 |
| *Sordariomycetes* | Main *vs.* S | 0.019 | 0.007 | 0.005 | 0.019 | 0.034 | 1 | 40559 |
|  | Main *vs.* M | -0.007 | 0.008 | -0.022 | -0.007 | 0.008 | 1 | 39689 |
|  | S *vs.* M | -0.026 | 0.006 | -0.038 | -0.026 | -0.015 | 1 | 40000 |
|  | Main *vs.* L | -0.027 | 0.011 | -0.048 | -0.027 | -0.006 | 1 | 40256 |
|  | S *vs.* L | -0.046 | 0.010 | -0.065 | -0.046 | -0.027 | 1 | 39739 |
|  | M *vs.* L | -0.020 | 0.010 | -0.040 | -0.020 | 0.000 | 1 | 39349 |
| *Mortierellomycetes* | Main *vs.* S | 0.168 | 0.024 | 0.120 | 0.168 | 0.215 | 1 | 40139 |
|  | Main *vs.* M | 0.157 | 0.024 | 0.109 | 0.157 | 0.205 | 1 | 40681 |
|  | S *vs.* M | -0.010 | 0.004 | -0.018 | -0.010 | -0.003 | 1 | 40000 |
|  | Main *vs.* L | 0.108 | 0.027 | 0.055 | 0.108 | 0.161 | 1 | 40480 |
|  | S *vs.* L | -0.060 | 0.012 | -0.084 | -0.060 | -0.035 | 1 | 39978 |
|  | M *vs.* L | -0.049 | 0.012 | -0.074 | -0.049 | -0.024 | 1 | 40463 |

**Table S7** Pearson correlation coefficients between the relative abundances of bacterial phyla or fungal classes and selected explanatory variables. Asterisks indicate statistical significance: * *P* ≤ 0.05, ** *P* ≤ 0.01, and ****P* ≤ 0.001.

| **Phyla or classes** | **Soil moisture** | **soil pH** | **SOC** | **Total N** | **Total P** | **soil C:N** | **soil C:P** | **soil N:P** |
| --- | --- | --- | --- | --- | --- | --- | --- | --- |
| *Actinobacteriota* | -0.59*** | -0.13 | 0.28*** | 0.03 | -0.18** | 0.34*** | 0.34*** | 0.19** |
| *Proteobacteria* | 0.65*** | 0.18** | 0.07 | 0.29*** | 0.21** | -0.24*** | -0.01 | 0.14* |
| *Acidobacteriota* | 0.16* | -0.21** | -0.25*** | -0.16* | 0.10 | -0.17* | -0.26*** | -0.22** |
| *Chloroflexi* | -0.32*** | 0.19** | -0.42*** | -0.51*** | -0.17* | 0.02 | -0.35*** | -0.45*** |
| *WPS-2* | -0.05 | -0.39*** | -0.11 | -0.09 | 0.04 | -0.05 | -0.13 | -0.13 |
| *Verrucomicrobiota* | 0.40*** | 0.17* | -0.07 | 0.07 | 0.10 | -0.16* | -0.13 | -0.04 |
| *Planctomycetota* | 0.08 | -0.07 | 0.03 | -0.01 | -0.09 | 0.08 | 0.09 | 0.09 |
| *Gemmatimonadota* | 0.19** | -0.24*** | -0.01 | 0.17* | 0.24*** | -0.19** | -0.11 | 0.00 |
| *Myxococcota* | 0.44*** | -0.13 | 0.04 | 0.14* | 0.12 | -0.12 | 0.04 | 0.15* |
| *Agaricomycetes* | -0.22** | -0.24*** | 0.07 | -0.14 | -0.10 | 0.26*** | 0.17* | 0.01 |
| *Tremellomycetes* | 0.30*** | -0.11 | 0.10 | 0.24*** | 0.13 | -0.15* | 0.04 | 0.16* |
| *Eurotiomycetes* | -0.45*** | 0.28*** | -0.10 | -0.25*** | -0.10 | 0.15* | -0.09 | -0.22** |
| *Sordariomycetes* | 0.41*** | 0.04 | 0.05 | 0.12 | 0.20** | -0.04 | -0.06 | -0.05 |
| *Mortierellomycetes* | 0.64*** | -0.05 | -0.03 | 0.30*** | 0.25*** | -0.36*** | -0.17* | 0.05 |
| *unclassfied_Rozellomycota* | -0.14* | -0.16* | 0.13 | 0.05 | -0.12 | 0.15* | 0.17* | 0.15* |
| *Leotiomycetes* | 0.06 | 0.28*** | -0.05 | -0.03 | -0.04 | -0.06 | -0.04 | 0.00 |
| *Geminibasidiomycetes* | -0.35*** | 0.11 | -0.23*** | -0.28*** | -0.14* | 0.02 | -0.16* | -0.21** |
| *Dothideomycetes* | -0.04 | 0.30*** | -0.22** | -0.11 | 0.1 | -0.22** | -0.31*** | -0.26*** |

**Table S8** Results of generalized additive models (GAMs) using non-parametric smoothers to identify the most important explanatory variables for microbial biomass, richness, and dissimilarity. The selected explanatory variables were based on significant accuracy importance (*P* < 0.05) after random forest classification (Figure S6).

| **Covariates** | **Bacterial biomass** | |  | **Bacterial richness** | |  | **Bacterial dissimilarity** | |
| --- | --- | --- | --- | --- | --- | --- | --- | --- |
|  | ***F* value** | ***P* value** |  | ***F* value** | ***P* value** |  | ***F* value** | ***P* value** |
| Area |  |  |  | 1.451 | 0.18 |  | 2.127 | <0.001 |
| Distance | 9.413 | 0.002 |  | 13.260 | <0.001 |  | 13.177 | <0.001 |
| Soil pH | 25.570 | <0.001 |  | 42.650 | <0.001 |  | 6.990 | <0.001 |
| Soil moisture |  |  |  | 7.286 | <0.001 |  |  |  |
| Nutrient quality |  |  |  | 3.482 | 0.009 |  | 9.882 | <0.001 |
| Microbial richness |  |  |  |  |  |  | 395.2 | <0.001 |
| Adjust R^2^ |  | 0.135 |  |  | 0.623 |  |  | 0.898 |
| Deviance explained (%) |  | 14.50% |  |  | 65.60% |  |  | 91.10% |
| REML |  | 557.36 |  |  | 1033.6 |  |  | -405.63 |
|  | Fungal biomass | |  | Fungal richness | |  | Fungal dissimilarity | |
|  | *F* value | *P* value |  | *F* value | *P* value |  | *F* value | *P* value |
| Area |  |  |  | 5.882 | 0.016 |  |  |  |
| Distance |  |  |  | 2.363 | 0.041 |  | 15.770 | <0.001 |
| Soil pH |  |  |  | 4.063 | 0.045 |  |  |  |
| Soil moisture | 15.730 | <0.001 |  |  |  |  |  |  |
| Nutrient quantity | 11.480 | <0.001 |  | 3.708 | 0.003 |  | 2.982 | 0.043 |
| Microbial richness |  |  |  |  |  |  | 165.180 | <0.001 |
| Adjust R^2^ |  | 0.085 |  |  | 0.253 |  |  | 0.574 |
| Deviance explained (%) |  | 9.53% |  |  | 30.30% |  |  | 58.60% |
| REML |  | 147.54 |  |  | 877.46 |  |  | -344.89 |


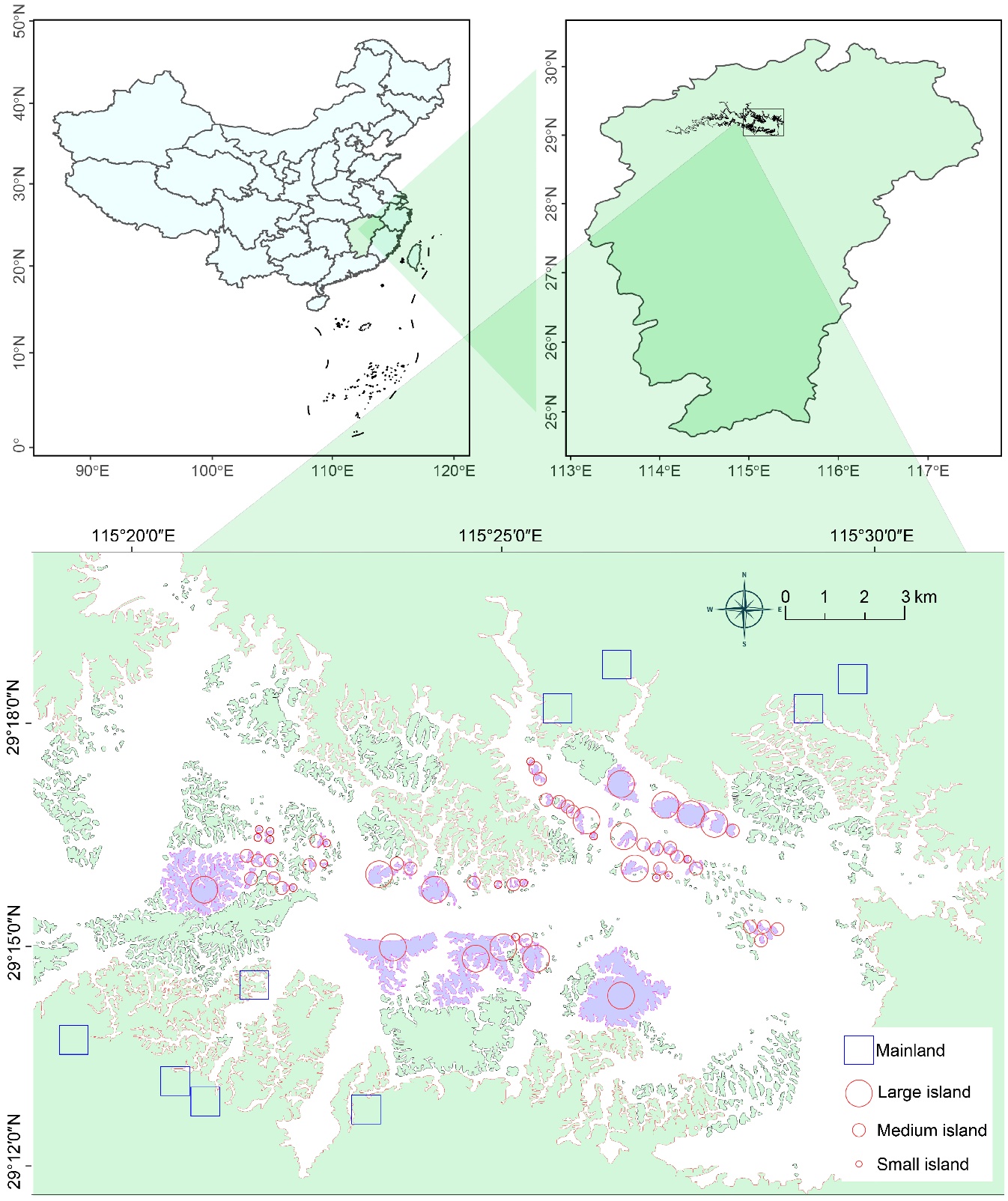


**Figure S1** Locations of the 61 land-bridge islands and 9 mainland sites in the Zhelin Reservoir, Jiangxi Province, China. The sampled islands are indicated by small, medium, and large red circles (indicating small, medium, and large islands), and the sampled mainland sites are indicated by blue squares.

**Figure S2** Differences in island area, distance-to-mainland, perimeter, perimeter: area ratio, shape index, and NDVI among three land-area types (S, small islands; M, medium islands; L, large islands). Boxplots indicate the posterior distributions. Different letters indicate a credibly different posterior distribution among the three island types (Bayesian-ANOVA, 95% highest density intervals of the posterior difference between each two groups falls above or below zero).

**Figure S3** Rarefaction curves of bacterial (a) and fungal (b) ASV for the 210 samples.

**Figure S4** Microbial balance of bacterial and fungal genus in mainland and island samples.

**Figure S5** Relationships between microbial signatures and soil properties.

**Figure S6** Relationships between microbial richness and island area or distance to mainland.

**Figure S7** Structural equation model (SEM) analyses of the direct and indirect effects of habitat subdivision on bacterial and fungal richness, dissimilarity, and biomass (a-b) across the three island types. Soil SQ, soil substrate quantity; Soil SL, soil substrate quality. Arrows indicate significant (*P* ≤ 0.05) effects. Values associated with arrows represent standardized path coefficients; pathways without significant effects (*P* > 0.05) are not shown. Values associated with response variables in brackets indicate the proportion of variation (*R*^2^) explained by relationships with other variables.

**Figure S8** Random Forest mean predictor importance (percentage of increase of mean square error) of explanatory variables on bacterial biomass (a), bacterial richness (b), bacterial dissimilarity (c), fungal biomass (d), fungal richness (e), and fungal dissimilarity (f). The accuracy importance measure was computed for each tree and was averaged over the forest. Asterisks indicate statistical significance: *, *P* ≤ 0.05; **, *P* ≤ 0.01.
